# JOINT ASSOCIATIONS OF PHYSICAL ACTIVITY AND SLEEP DURATION WITH COGNITIVE AGING

**DOI:** 10.1093/geroni/igad104.0411

**Published:** 2023-12-21

**Authors:** Mikaela Bloomberg, Laura Brocklebank, Mark Hamer, Andrew Steptoe

**Affiliations:** University College London, London, England, United Kingdom; University College London, London, England, United Kingdom; University College London, London, England, United Kingdom; University College London, London, England, United Kingdom

## Abstract

Given the paucity of dementia treatments, identifying contributors to cognitive decline from midlife is important to delay onset of symptoms. Low physical activity (PA) and nightly sleep duration outside 6-8 hours are key interrelated factors thought to contribute to cognitive decline and dementia; how PA and sleep combine to influence cognitive ageing is not well-explored. We used linear mixed models to examine independent and joint associations of PA (low, high) and sleep duration (short [< 6 hours], optimal [6-8], long [>8]) with 10-year cognitive trajectories in cognitively-healthy adults aged ≥50 years from the English Longitudinal Study of Ageing (N=8958). We found low PA (p< 0.001) and suboptimal sleep (p=0.01) were independently associated with worse cognitive performance; short sleep was also associated with faster cognitive decline (p=0.04). At baseline, high PA/optimal sleepers had higher cognitive scores than all sleep categories in the low PA category (e.g. difference high PA/optimal-low PA/short at age 50=0.14 [0.05, 0.24] standard deviations), with no difference in cognitive scores between sleep categories within the high PA category. Differences remained consistent over 10 years of follow-up, except high PA/short sleepers who declined faster than high PA/optimal sleepers, such that scores at 10 years were commensurate with low PA (difference high PA/optimal-high PA/short=0.20 [0.08, 0.33]; high PA/optimal-low PA/short=0.22 [0.11, 0.34]). Baseline cognitive benefits afforded by high PA were insufficient to ameliorate rapid cognitive decline associated with short sleep. While WHO identify PA as a key target for maintaining cognitive health, PA interventions may be ineffective without considering sleep habits.

